# Angioimmunoblastic T-cell lymphoma with secondary hemophagocytic lymphohistiocytosis initially misdiagnosed as classical Hodgkin lymphoma: A case report

**DOI:** 10.1097/MD.0000000000050979

**Published:** 2026-10-02

**Authors:** Rui Li, Yong Chen, Xuefen Liu, Yuying Gao, Nengping Zhu, Changyu Deng, Yue Li

**Affiliations:** a Department of Oncology, The People’s Hospital of Rongchang District, Chongqing, China; b Department of Cardiology, The People’s Hospital of Rongchang District, Chongqing, China.

**Keywords:** angioimmunoblastic T-cell lymphoma, dexamethasone, Epstein–Barr virus, etoposide, hemophagocytic lymphohistiocytosis

## Abstract

**Rationale:**

Angioimmunoblastic T-cell lymphoma (AITL), classified as nodal T-follicular helper cell lymphoma, angioimmunoblastic type, is an aggressive lymphoma frequently complicated by immune dysregulation. Owing to overlapping histopathological features, particularly Hodgkin/Reed–Sternberg–like cells, AITL may be initially misdiagnosed as Hodgkin lymphoma, leading to delayed recognition and inappropriate management.

**Patient concerns:**

A 72-year-old man presented with progressive fatigue, recurrent fever, severe pancytopenia, and splenomegaly after an outside diagnosis of Hodgkin lymphoma based on inguinal lymph node biopsy.

**Diagnoses:**

Laboratory findings revealed hypertriglyceridemia and marked hyperferritinemia, consistent with secondary hemophagocytic lymphohistiocytosis (HLH). Reevaluation of archived lymph node tissue demonstrated Epstein–Barr virus–encoded RNA positivity, T-cell receptor gene rearrangement, and an immunophenotype diagnostic of nodal T-follicular helper cell lymphoma, angioimmunoblastic type with scattered Hodgkin/Reed–Sternberg–like cells. Plasma Epstein–Barr virus DNA was detected at low levels. The disease was staged as Lugano stage III with B symptoms and classified as high risk by the International Prognostic Index.

**Interventions:**

HLH-directed therapy with etoposide and dexamethasone was initiated for rapid disease control, followed by cyclophosphamide, doxorubicin, vincristine, and prednisone chemotherapy for lymphoma treatment.

**Outcomes:**

Partial remission was achieved after 6 cycles of cyclophosphamide, doxorubicin, vincristine, and prednisone chemotherapy. The patient later experienced disease progression with clinically suspected recurrent HLH despite salvage therapy and died on March 29, 2025.

**Lessons:**

This case underscores the diagnostic pitfalls of AITL complicated by HLH and highlights the importance of early pathological reassessment and a stepwise treatment approach prioritizing control of hyperinflammation in patients with discordant clinical and pathological findings.

## 1. Introduction

Angioimmunoblastic T-cell lymphoma (AITL) is a rare and aggressive subtype of peripheral T-cell lymphoma characterized by systemic inflammatory manifestations and immune dysregulation.^[1,2]^ Owing to its heterogeneous clinical presentation and overlapping histopathological features, AITL is frequently misdiagnosed as other lymphoid malignancies, most notably Hodgkin lymphoma, which may result in delayed or inappropriate treatment.^[3]^

Hemophagocytic lymphohistiocytosis (HLH) is a life-threatening hyperinflammatory syndrome caused by uncontrolled immune activation and is commonly associated with lymphoid malignancies, including AITL. HLH typically presents with clinical manifestations such as persistent fever and splenomegaly and is accompanied by characteristic hematologic, biochemical, immunologic, and other diagnostic abnormalities, including cytopenias, hypertriglyceridemia and/or hypofibrinogenemia, hyperferritinemia, hemophagocytosis, reduced or absent natural killer cell activity, and elevated soluble CD25 levels.^[4,5]^ When HLH develops secondary to lymphoma, its clinical presentation may closely resemble severe infection or sepsis, further complicating diagnostic evaluation and delaying oncologic reassessment.

The coexistence of AITL and HLH presents substantial diagnostic challenges, as excessive immune activation and prominent histiocytic infiltration may obscure the underlying lymphoma, resulting in diagnostic delay or misclassification.^[6]^

## 2. Case presentation

### 2.1. Initial presentation

In February 2022, a 72-year-old man initially presented with a gradually enlarging, painless mass in the right inguinal region and underwent excisional lymph node biopsy at an outside institution. According to the original pathology report, the findings were interpreted as classical Hodgkin lymphoma (cHL).

The initial diagnosis was supported by immunohistochemical findings showing positivity for CD20, PAX-5, CD30, CD15, CD21 and CD23 (highlighting expanded follicular dendritic cell networks), Bcl-2, and Epstein–Barr virus (EBV) latent membrane protein 1, with a high Ki-67 proliferation index, whereas CD3, CD5, CD10, TdT, PD-1, and ALK were reported as negative. The pathological interpretation at that time was based on the contemporaneous pathology report.

Following the initial diagnosis, the patient did not initiate lymphoma-directed chemotherapy because of fear of chemotherapy and instead sought treatment with traditional Chinese medicine. During this interval, no structured oncologic reassessment was performed. Approximately 9 months later, in early 2023, the patient gradually developed progressive upper abdominal distension aggravated after meals, accompanied by intermittent cough and exertional fatigue, and was subsequently admitted to our department for further evaluation and management.

### 2.2. Diagnostic workup

On February 20, 2023, contrast-enhanced computed tomography of the chest and abdomen revealed extensive lymphadenopathy involving the bilateral axillary, mediastinal, retroperitoneal, pelvic, and bilateral inguinal regions, with relatively homogeneous enhancement, accompanied by marked splenomegaly (Fig. 1A and 1B).

**Figure 1.**
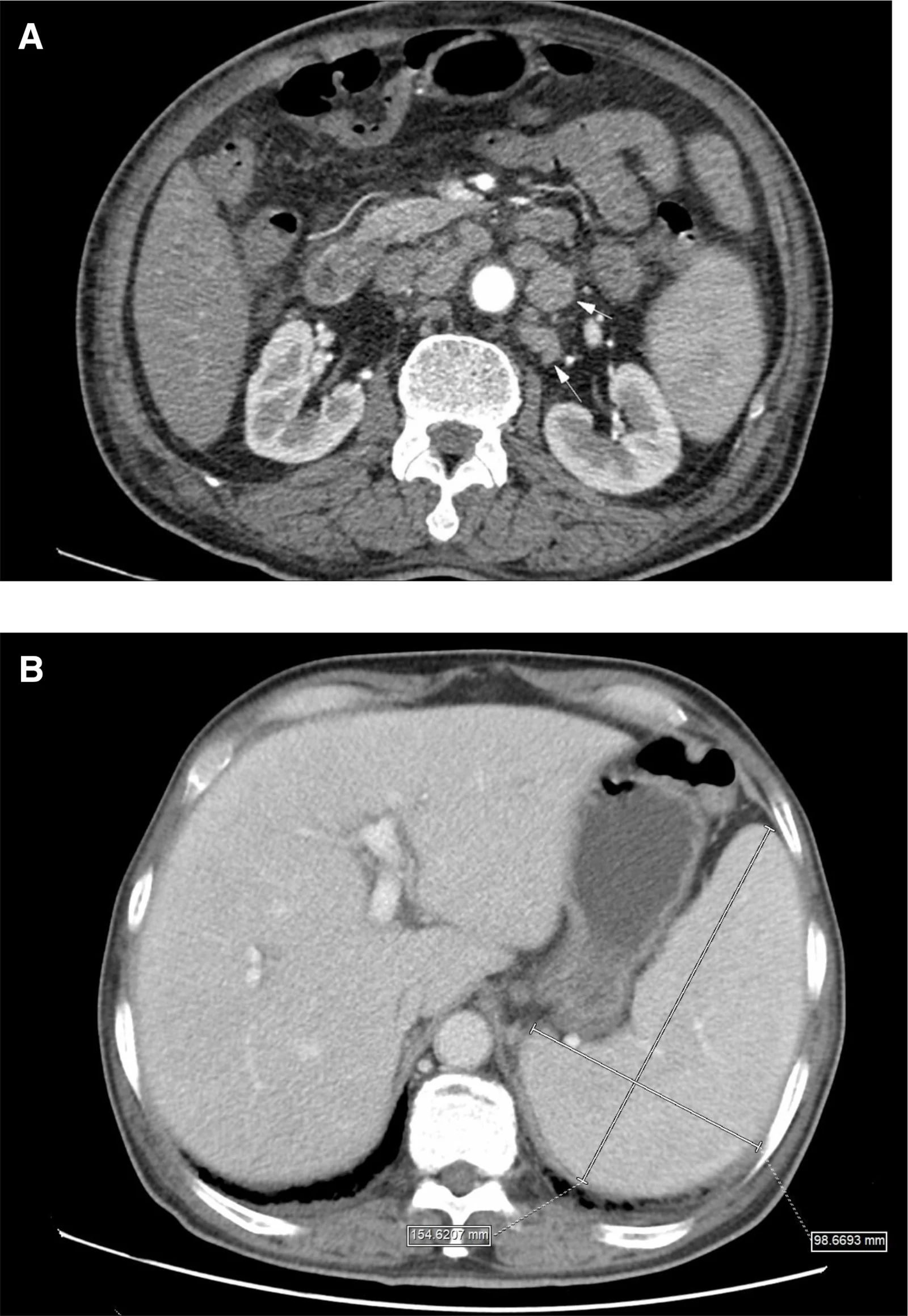
Baseline contrast-enhanced abdominal computed tomography (CT) performed on February 20, 2023. (A) Arterial-phase CT demonstrating multiple enlarged retroperitoneal lymph nodes (arrows). (B) CT image showing marked splenomegaly, with splenic dimensions of approximately 154.6 × 98.7 mm.

Bone marrow aspiration performed on March 8, 2023, revealed a hypercellular marrow with abundant megakaryocytes and active granulocytic and erythroid proliferation. No definite hemophagocytosis was identified on bone marrow smears at that time. During hospitalization, serial complete blood counts documented progressive and severe pancytopenia, accompanied by persistent fever. Further laboratory evaluation demonstrated marked hypertriglyceridemia (7.64 mmol/L; reference range, 0–2.3 mmol/L), hyperferritinemia (993.30 µg/L; reference range, 23.9–336.2 µg/L), and elevated soluble CD25 receptor levels (8074 U/mL; reference range, 223–710 U/mL); other HLH-related parameters were not serially available owing to limited testing during the acute critical phase. Dynamic changes in hematologic and inflammatory parameters during diagnostic evaluation and early management (March–April 2023) are summarized in Table 1.

**Table 1:** Dynamic changes in hematologic, biochemical, and inflammatory parameters during the diagnostic evaluation and early management of secondary HLH.

Items	Reference range	2023-03-07	2023-03-13	2023-03-17	2023-03-21	2023-03-24	2023-03-29	2023-04-03	2023-04-10	2023-04-14
WBC (10^9^/L)	4–9.15	2.2	1.6	2.1	1.1	3.6	1.6	1.3	3	6.1
PMN (10^9^/L)	2–7.7	1.75	1.05	1.87	0.8	3.3	1.26	0.94	2.55	2.87
PLT (10^9/^L)	85–303	1	5	16	25	30	46	60	77	100
RBC (10^12^/L)	4–5.5	2.49	2.1	2.49	2.45	2.67	2.77	3.17	3.29	3.14
Hb (g/L)	120–160	76	56	68	67	76	83	95	105	98
HCT (%)	38–50.8	23.2	19.3	23.4	22.2	24.9	26.9	31.7	33.1	31.4
TG (mmol/L)	0–2.3	nd	7.64	nd	nd	nd	nd	nd	nd	nd
ALT (U/L)	9–50	59.50	27.10	nd	nd	19.30	nd	24.60	14.20	nd
AST (U/L)	15–40	58.60	42.10	nd	nd	18.80	nd	21.10	19.00	nd
GGT (U/L)	10–60	398.30	271.10	nd	nd	212.50	nd	178.90	147.80	nd
ALB (g/L)	35–55	29.50	26.20	nd	nd	34.70	nd	42.30	40.00	nd
Fer (µg/L)	23.9–336.2	nd	993.3	nd	nd	nd	nd	nd	nd	nd
sCD25R (U/mL)	223–710	nd	8074	nd	nd	nd	nd	nd	nd	nd
LDH (U/L)	109–245	271	nd	nd	nd	nd	nd	nd	nd	nd

ALB = albumin, ALT = alanine aminotransferase, AST = aspartate aminotransferase, Fer = ferritin, GGT = γ-glutamyltransferase, Hb = hemoglobin, HCT = hematocrit, LDH = lactate dehydrogenase, nd = not determined (test not performed or data unavailable at that time point), PLT = platelet count, PMN = neutrophil count, RBC = red blood cell count, sCD25R = soluble CD25 receptor, TG = triglyceride, WBC = white blood cell count.

Twenty unstained slides from the original right inguinal lymph node biopsy were submitted for pathological consultation and underwent further immunohistochemical, Epstein–Barr virus–encoded RNA (EBER) in situ hybridization, and molecular evaluation. In situ hybridization demonstrated EBER positivity, with scattered expression in large cells. Immunoglobulin heavy-chain gene rearrangement was negative, whereas T-cell receptor (TCR) gene rearrangement was positive (3 of 6 tubes positive: TCR β Vβ–Jβ1/2, Dβ–Jβ1/2; TCRγ Vγ1–8, Vγ10 with multiple Jγ regions). Immunohistochemistry revealed CD21 positivity with disrupted residual follicular dendritic cell networks, CD2+, CD3+, CD5+, partial CD7+, CD4 predominance over CD8, focal PD-1 positivity, Bcl-6 positivity in a subset of cells (~30%), scattered CD30-positive Hodgkin/Reed–Sternberg (HRS)–like cells (~3%, moderate to strong intensity), CD15 negativity, CD20 positivity in residual follicular-zone cells and HRS-like cells, weak PAX-5 expression in HRS-like cells, Bob.1 negativity, Oct-2 positivity in large cells, and a Ki-67 proliferation index of approximately 40%. Compared with the original outside pathology report, which described CD3, CD5, and PD-1 as negative and CD15 as positive, the subsequent consultation reported CD3 and CD5 positivity, focal PD-1 positivity, and CD15 negativity. Because both assessments concerned material from the same original biopsy, these discrepancies should not be interpreted as a proven biological conversion of marker expression. Complete technical details of the initial outside immunohistochemical studies were unavailable; therefore, the relative contributions of differences in staining procedures, tissue sections, technical factors, or pathological interpretation could not be reliably determined.

Based on the integrated histopathological, immunophenotypic, and molecular findings, a diagnosis of nodal T-follicular helper (TFH) cell lymphoma, angioimmunoblastic type (AITL; according to the 2022 World Health Organization classification), with HRS-like cells was established.

The patient fulfilled at least 5 HLH-2004 diagnostic criteria, including persistent fever, progressive pancytopenia affecting all 3 hematopoietic lineages, marked splenomegaly, hypertriglyceridemia, hyperferritinemia, and markedly elevated soluble CD25 levels, thereby supporting the diagnosis of secondary hemophagocytic lymphohistiocytosis (HLH).

During the diagnostic evaluation, plasma EBV DNA testing performed on April 17, 2023, detected low-level viremia of 4.46 × 10^2^ copies/mL, which supported EBV-associated immune activation.

Based on the extent of nodal involvement and systemic symptoms, the disease was staged as Lugano stage III with B symptoms and classified as high risk according to the International Prognostic Index.

### 2.3. Management

Given the diagnosis of secondary HLH and the presence of a life-threatening hyperinflammatory state, HLH-directed therapy with etoposide combined with dexamethasone was initiated on March 15, 2023, and continued for approximately 1 month. Following treatment, the patient experienced resolution of fever, improvement in cytopenias, and overall clinical stabilization.

Considering the patient’s age and general condition, autologous hematopoietic stem cell transplantation was not considered appropriate. Chidamide is an orally active, subtype-selective histone deacetylase inhibitor with documented clinical activity in relapsed or refractory peripheral T-cell lymphoma, including AITL.^[7]^ Given the diagnosis of AITL and the available clinical activity data for chidamide in this disease context, the treating team considered chidamide as part of the planned lymphoma-directed strategy. A treatment strategy consisting of 4 to 6 cycles of cyclophosphamide, doxorubicin, vincristine, and prednisone (CHOP) chemotherapy combined with chidamide, followed by chidamide maintenance for 1 to 2 years, was formulated. The overall unfavorable prognosis of AITL was fully discussed with the patient and his family. However, due to financial constraints, chidamide was not administered during the initial treatment phase, and the patient received CHOP alone.

### 2.4. Clinical course

From April 18 to August 2, 2023, the patient completed 6 cycles of CHOP chemotherapy. Interim response assessment, performed on July 12, 2023, using contrast-enhanced computed tomography of the chest and abdomen, demonstrated radiologic partial remission (Fig. 2A and 2B). Post-treatment bone marrow reassessment was not available; therefore, the status of marrow involvement and hematologic remission could not be further evaluated, which represents a limitation of this case.

**Figure 2.**
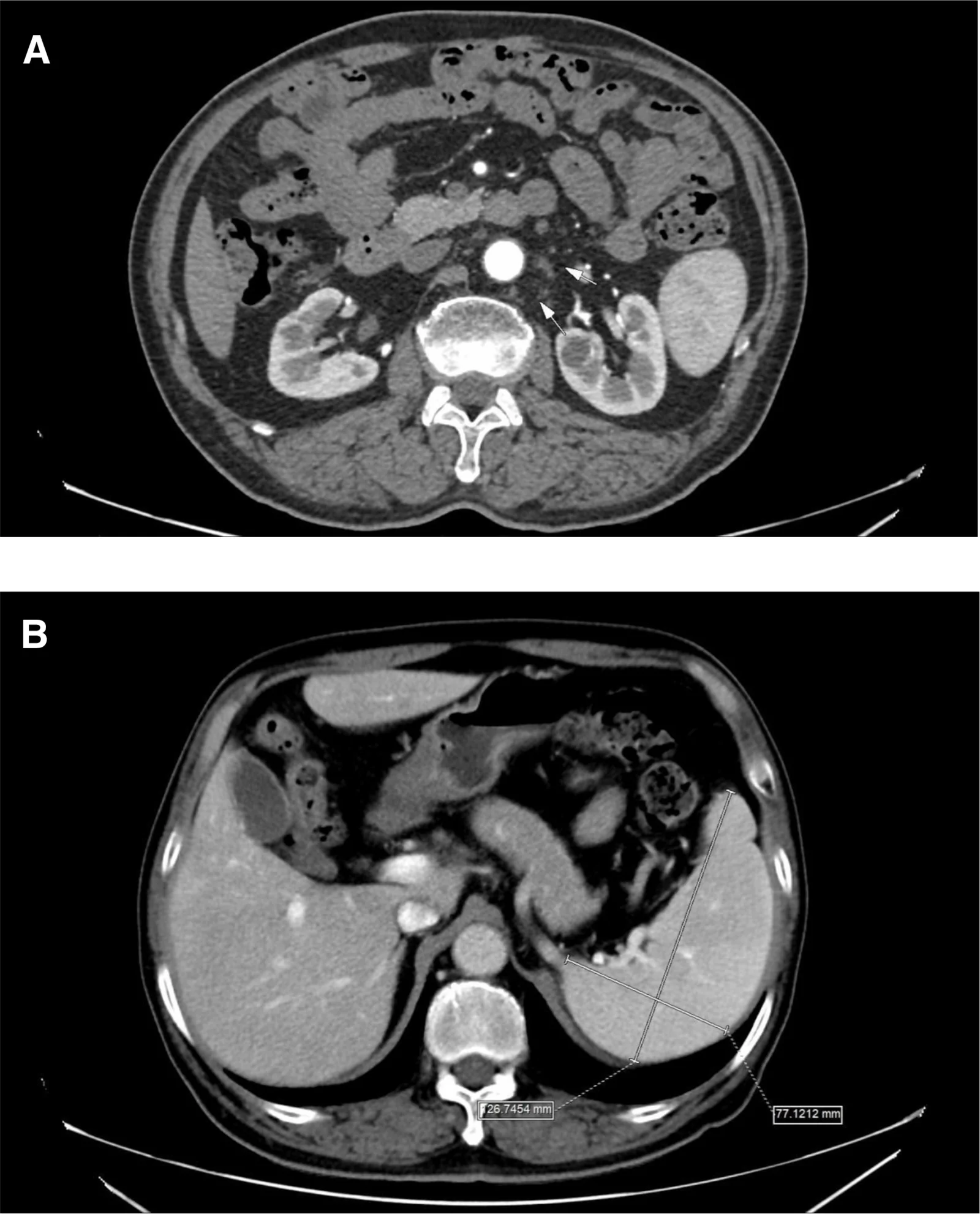
Follow-up contrast-enhanced abdominal computed tomography (CT) performed on July 12, 2023. (A) Arterial-phase CT showing marked regression of retroperitoneal lymphadenopathy compared with baseline imaging (arrows). (B) CT image demonstrating decreased splenic size, measuring approximately 126.7 × 77.1 mm.

From August 2023 to July 2024, the patient was followed intermittently in the outpatient setting. His general condition remained relatively stable without recurrent fever or overt manifestations of HLH, although no scheduled imaging reassessment was performed during this interval.

In August 2024, the patient developed progressive exertional dyspnea, fatigue, abdominal distension, anorexia, and recurrent cytopenias. Imaging studies revealed widespread progression of lymphadenopathy involving the bilateral cervical, axillary, mediastinal, hilar, retroperitoneal, iliac, and bilateral inguinal regions, as well as newly developed abdominopelvic effusion. Recurrent HLH was clinically suspected in the setting of progressive lymphoma and recurrent cytopenias; however, a complete reassessment of the HLH-2004 diagnostic criteria was not available at that time. Etoposide combined with dexamethasone was reinitiated on August 3, 2024, resulting in transient clinical stabilization.

Subsequent salvage chemotherapy with a gemcitabine, dexamethasone, and cisplatin regimen was administered, followed by dose-adjusted cycles due to grade IV myelosuppression. Chidamide (20 mg orally twice weekly) was later introduced and continued as maintenance therapy, leading to symptomatic relief.

Two days prior to the final admission, the patient presented with cough and dyspnea without fever or chest pain. Despite supportive care, his condition progressively deteriorated, and he died on March 29, 2025.

## 3. Discussion

AITL is a rare subtype of peripheral T-cell lymphoma, accounting for approximately 1% to 2% of non-Hodgkin lymphomas, and is associated with an unfavorable prognosis.^[8]^ Its clinical manifestations are often heterogeneous, and the underlying histopathological features may overlap with those of other lymphoproliferative disorders, making accurate diagnosis challenging. In particular, AITL may be misdiagnosed as cHL when EBV-positive HRS-like B cells are prominent and the underlying neoplastic TFH cell component is subtle or obscured, with partial expression of B-cell–associated markers, thereby closely mimicking the immunophenotypic features of cHL.^[9]^ In the present case, the initial diagnosis of cHL was revised after further pathological and immunohistochemical evaluation, highlighting the importance of diagnostic reconsideration in atypical or refractory cases.

The differential diagnosis of AITL is particularly important when prominent EBV-associated B-cell proliferation and HRS-like cells obscure the underlying neoplastic T-cell population. In such cases, diagnosis should not rely on HRS-like cells alone but should integrate lymph node architecture, TFH-associated immunophenotypic findings, follicular dendritic cell network alterations, EBER findings, and molecular evidence of T-cell clonality. Relevant differential diagnoses include cHL, peripheral T-cell lymphoma not otherwise specified, other nodal TFH-cell lymphomas, and EBV-associated B-cell lymphoproliferative processes. Flow-cytometric results were not available in the accessible medical records for this retrospective case. When AITL is suspected, timely collection of fresh tissue should be considered whenever feasible to permit flow-cytometric immunophenotyping, which may provide useful complementary diagnostic information.^[8,9]^

Lymphoma-associated HLH represents a severe and life-threatening complication characterized by excessive immune activation and systemic inflammation. Clinical manifestations commonly include persistent fever and splenomegaly, whereas laboratory and other diagnostic abnormalities may include cytopenias, hypertriglyceridemia and/or hypofibrinogenemia, hyperferritinemia, elevated soluble CD25 levels, and other HLH-related findings.^[10,11]^ According to the HLH-2004 diagnostic guidelines, the diagnosis of HLH is generally based on an integrated assessment of clinical features, laboratory abnormalities, and relevant histopathologic findings, with the diagnosis established when multiple diagnostic criteria are fulfilled.^[11]^ The rapid progression and high mortality associated with HLH continue to pose major challenges to early recognition and timely intervention.^[12]^ Because of its nonspecific presentation and aggressive clinical course, lymphoma-associated HLH is frequently recognized only after substantial disease progression, which can adversely affect prognosis.^[13,14]^

EBV infection plays an important role in the pathogenesis of both AITL and HLH. EBV is a well-established risk factor for lymphoid malignancies and has been strongly associated with the development of HLH. Elevated EBV-DNA levels have been shown to correlate with increased disease activity and poor prognosis in patients with lymphoma-associated HLH.^[15]^ Therefore, EBV-driven immune dysregulation may represent a key pathogenic link between AITL and secondary HLH.

Beyond biological complexity, this case also illustrates the potential clinical consequences of prolonged intervals without structured oncologic reassessment in aggressive hematologic malignancies. This observation underscores the importance of timely reassessment and continuity of care in clinical practice.

From a therapeutic perspective, early recognition of HLH and timely initiation of appropriate treatment are critical for improving outcomes. In this patient, treatment with etoposide and dexamethasone resulted in marked clinical and hematologic improvement, consistent with their established role in the management of lymphoma-associated HLH. Following stabilization, subsequent CHOP chemotherapy achieved partial remission, indicating that timely control of hyperinflammation may allow for effective treatment of the underlying lymphoma. Nevertheless, the diagnostic and therapeutic challenges posed by AITL complicated by HLH remain substantial, underscoring the need for heightened clinical awareness and further research.

A further limitation of this case is that the original biopsy and subsequent pathological consultation were performed at outside institutions. Despite our efforts to obtain the original high-resolution pathological image files suitable for publication, these images could not be retrieved and, therefore, were not available for inclusion in the manuscript.

From a general clinical perspective, unexplained fever accompanied by progressive cytopenias and splenomegaly – particularly when clinical features are discordant with initial pathological findings – should prompt early reconsideration of the underlying diagnosis and evaluation for secondary HLH. Timely pathological reevaluation and early initiation of HLH-directed therapy may help stabilize life-threatening hyperinflammation and facilitate subsequent treatment of the underlying lymphoma.

**Article highlights**

- AITL is a rare and aggressive subtype of peripheral T-cell lymphoma with heterogeneous clinical and pathological features, which may lead to frequent misdiagnosis.
- Secondary HLH can further complicate the clinical presentation of AITL and obscure the underlying malignancy, resulting in delayed diagnosis and treatment.
- This case highlights the diagnostic challenges posed by AITL complicated by HLH, particularly when initial pathological findings mimic cHL.
- Delayed oncologic reassessment may contribute to diagnostic delay and disease progression in aggressive hematologic malignancies.
- Timely pathological reevaluation and early initiation of HLH-directed therapy are essential in lymphoma-associated hemophagocytic lymphohistiocytosis.

## Acknowledgments

The authors are grateful to the patient for his cooperation. The authors also thank the Departments of Oncology and Cardiology of The People’s Hospital of Rongchang District and Wanling Town Health Center for their support in clinical care, case documentation, and manuscript preparation.

## Author contributions

**Data curation:** Rui Li, Yue Li.

**Formal analysis:** Rui Li, Yue Li, Yong Chen, Xuefen Liu.

**Resources:** Rui Li, Yong Chen, Nengping Zhu, Changyu Deng, Xuefen Liu, Yue Li.

**Supervision:** Yuying Gao, Xuefen Liu.

**Writing – original draft:** Rui Li.

**Writing – review & editing:** Rui Li, Yue Li.
